# Supplementary material for: Structural basis for the allosteric modulation of rhodopsin by nanobody binding to its extracellular domain
Source: Nat Commun. 2023 Aug 25;14:5209. doi: 10.1038/s41467-023-40911-9 (PMC10457330; doi:10.1038/s41467-023-40911-9)
Supplement: Supplementary file 1 — Supplementary Information [file 41467_2023_40911_MOESM1_ESM.pdf]

SUPPLEMENTARY INFORMATION

Title

Structural basis for the allosteric modulation of rhodopsin by nanobody binding to its extracellular domain

Contents:

Supplementary Figures 1-8

Supplementary Table 1

Supplementary References

Supplementary Figures

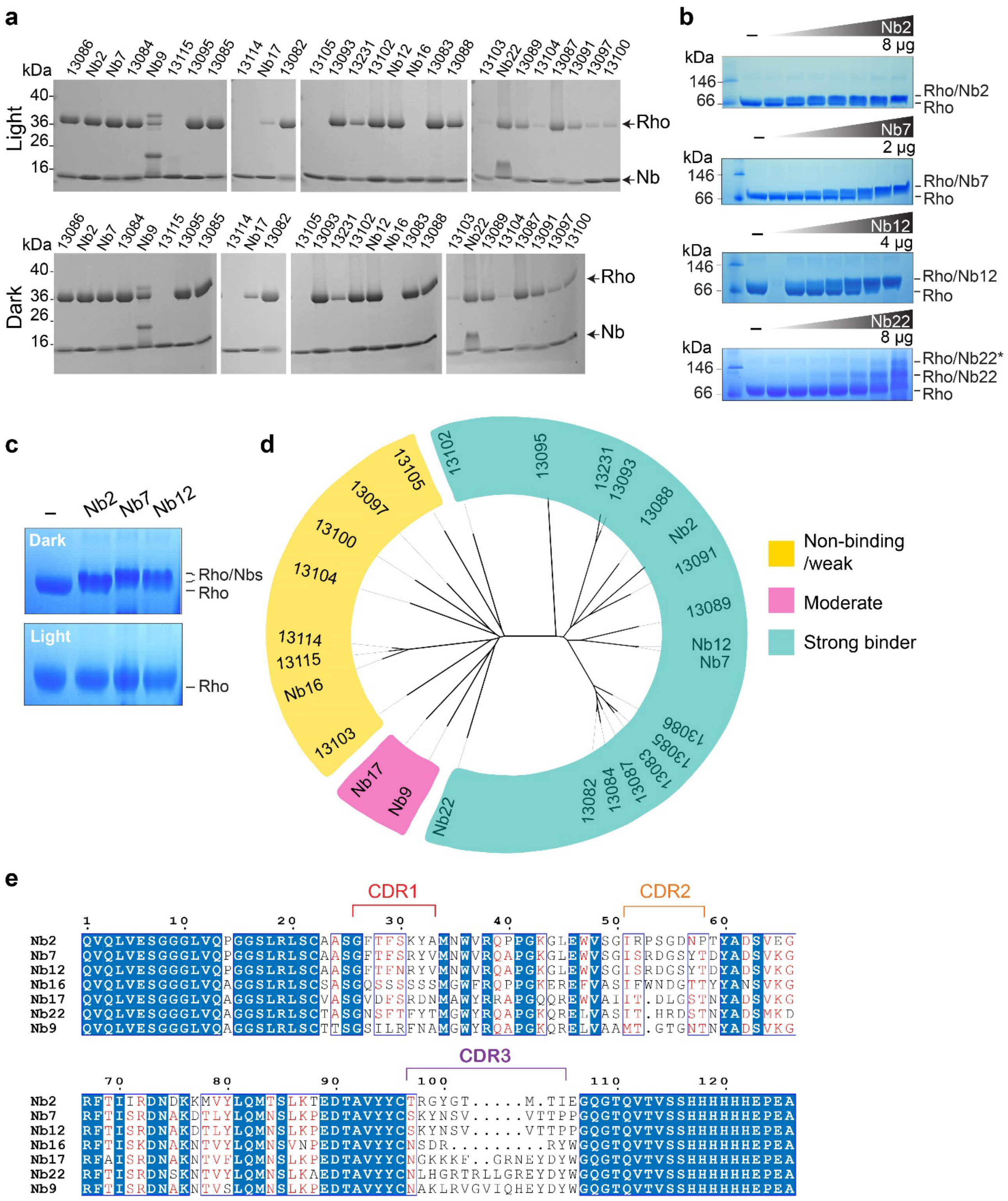

Supplementary Fig. 1. **Development of Nbs that bind to ground-state and photoactivated bRho. a** Ni- NTA co-purification of His-tagged Nbs with bRho in the dark or under light. Eluted bRho/Nb complexes were analyzed by Coomassie-stained SDS-PAGE. The experiment was carried out two times with similar results. **b** Blue native polyacrylamide gel electrophoresis (BN- PAGE), showing gel shifts of native bRho (3 μg) with two-fold increasing amounts of Nbs (2, 4, 8 μg), followed by BN-PAGE in the dark. Asterisk (*) indicates supershift of Nb22. **c** After incubation in the dark, Rho samples with and without Nbs were subjected to BN-PAGE either in the dark or light. In **b,c**, BN-PAGE experiments were carried out three times with similar results. **d** Unrooted phenogram for Nbs. The initial multiple sequence alignment and phylogenetic tree of the Nbs was obtained from Clustal Omega [1], and the final unrooted tree representation was drawn with iTOLS [2]. Nbs were classified into three groups, based on the binding results from **a-c**. **e** Multiple sequence alignment of Nb clones that were used in this study by *Clustal Omega* [3] and *ESPript* 3.0 [4]. Identical residues are shaded in blue. Conserved and similar residues are framed in blue and labeled in red letters, respectively. Complementarity Determining Regions (CDRs) are indicated in red, orange, and purple, respectively. Source data for panels **a-c** are provided as a Source Data file.

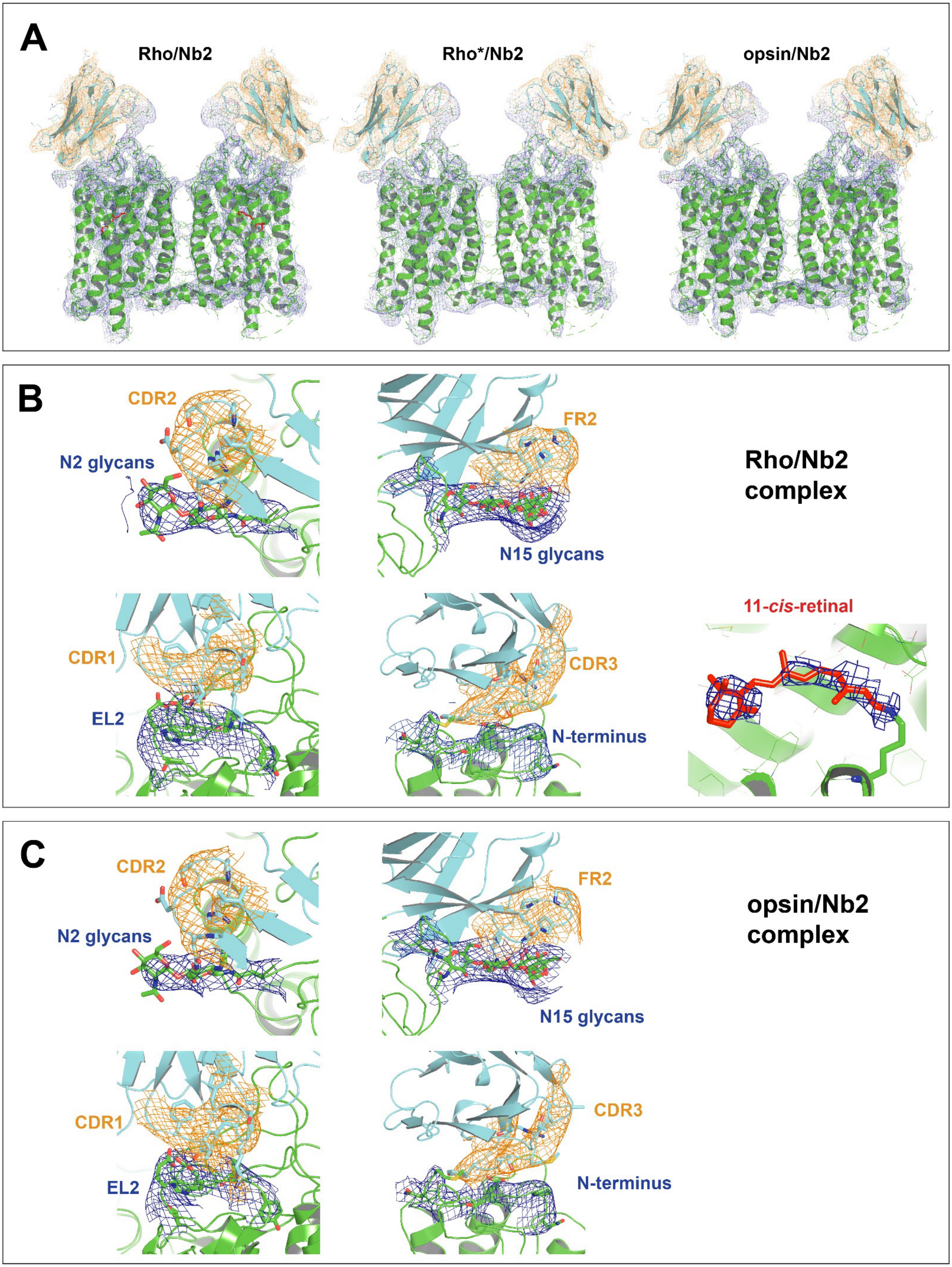
A
Rho/Nb2
Rho*/Nb2
opsin/Nb2
B
CDR2
N2 glycans
FR2
N15 glycans
Rho/Nb2 complex
CDR1
EL2
CDR3
N-terminus
11-cis-retinal
C
CDR2
N2 glycans
FR2
N15 glycans
opsin/Nb2 complex
CDR1
EL2
CDR3
N-terminus

Supplementary Fig. 2. **Representative $2F_o$-$2F_c$ electron density maps contoured at 1σ**. A) $2F_o$-$2F_c$ maps for the three crystal structures in this work. B) Electron density map for the interface region of bRho/Nb2 and for 11-*cis*-retinal. (C) Electron density map for the interface area between apo-opsin and Nb2.

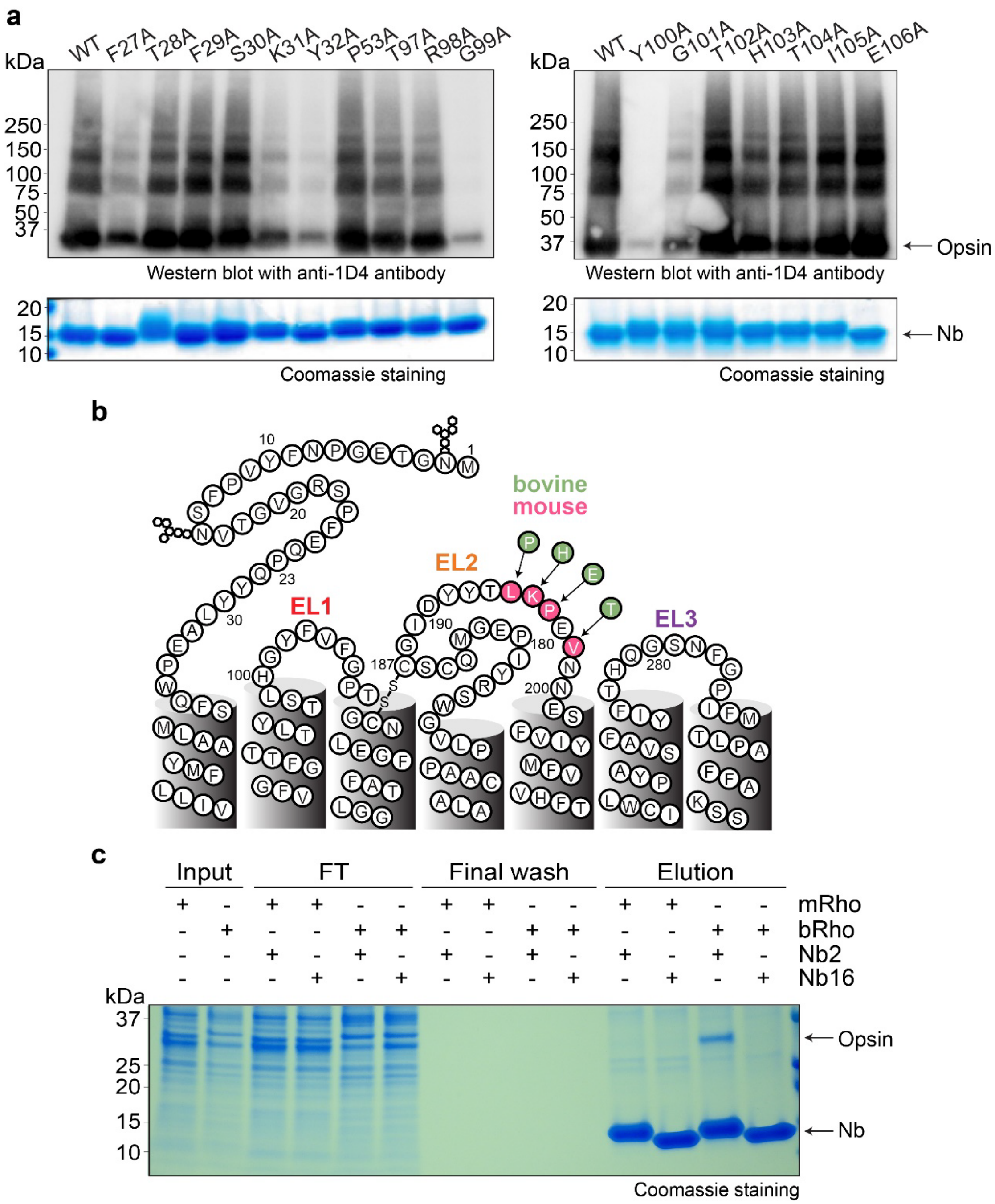

Supplementary Fig. 3. **Alanine scanning mutagenesis and species specificity of Nb2. a** Alanine scanning mutagenesis of the CDR1 and CDR3 regions of Nb2. Co-IP of the resultant mutants with bROS were analyzed by western blotting with anti-Rho 1D4 antibody and Coomassie blue stain to detect opsins and Nbs, respectively. **b** A secondary-structure model of mRho showing extracellular loops in the extracellular (intradiscal) side with N-

linked glycosides attached to Asn^{2} and Asn^{15}. The scheme shows the replacement on EL2 of murine Rho residues (pink) with bovine Rho residues (green). **c** SDS-PAGE stained with Coomassie blue showing Co-IP of either mRho or bRho with Nb2 or Nb16 (negative control) in the light. Data are representative of three biologically independent replicates. Source data for panels **a** and **c** are provided as a Source Data file.

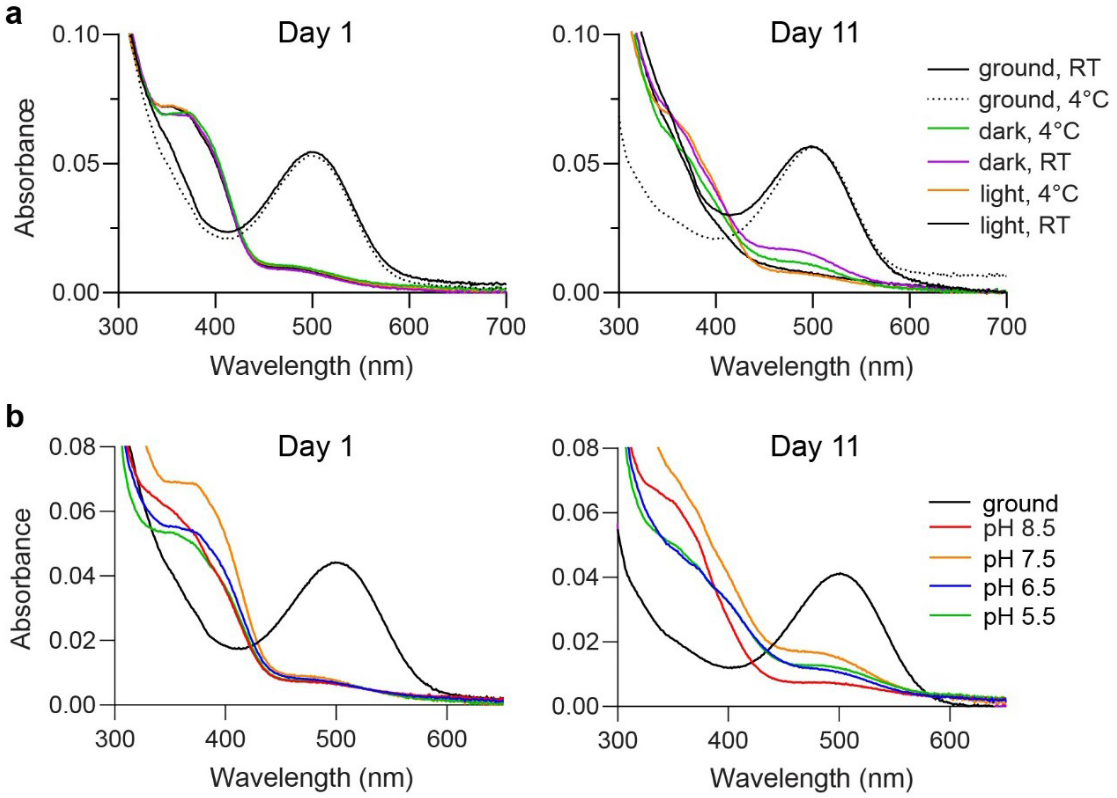

Supplementary Fig. 4. **Kinetic profiling of bRho alone under different light/dark, temperature, and pH conditions. a,b** Ground-state bRho was illuminated for 5 min, followed by UV-Vis spectroscopic measurements under light/dark and different temperatures at pH 7.5 (**a**) or different pHs in the dark at room temperature (**b**). Representative UV-Vis absorbance spectra of bRho under experimental conditions measured at day 1 and day 11. Source data for panels **a** and **b** are provided as a Source Data file.

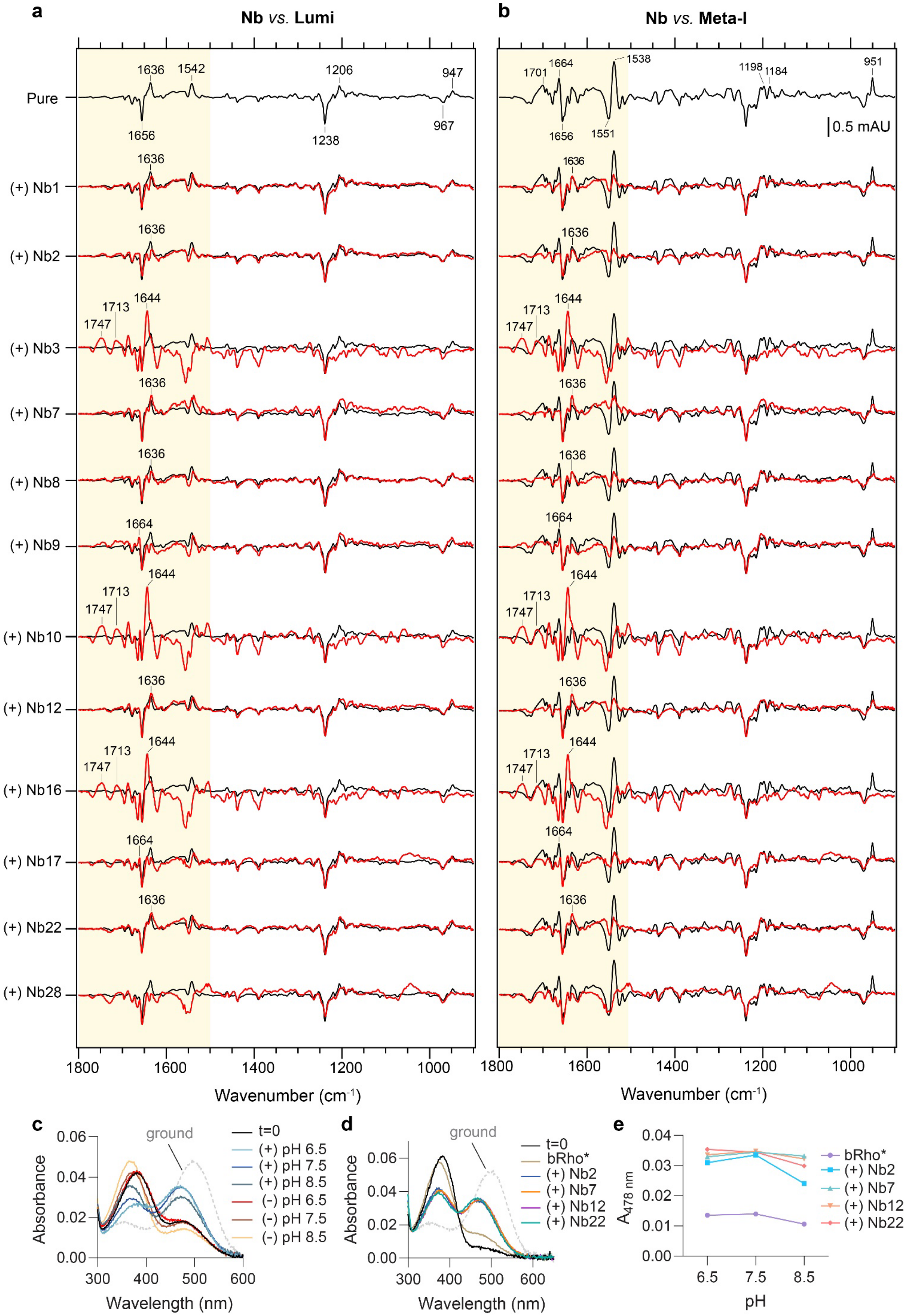
a
Nb vs. Lumi
b
Nb vs. Meta-I
Pure
(+) Nb1
(+) Nb2
(+) Nb3
(+) Nb7
(+) Nb8
(+) Nb9
(+) Nb10
(+) Nb12
(+) Nb16
(+) Nb17
(+) Nb22
(+) Nb28
0.5 mAU
Wavenumber (cm-1)
c
Absorbance
Wavelength (nm)
ground
t=0
(+) pH 6.5
(+) pH 7.5
(+) pH 8.5
(-) pH 6.5
(-) pH 7.5
(-) pH 8.5
d
Absorbance
Wavelength (nm)
ground
t=0
bRho*
(+) Nb2
(+) Nb7
(+) Nb12
(+) Nb22
e
A478 nm
pH
bRho*
(+) Nb2
(+) Nb7
(+) Nb12
(+) Nb22

Supplementary Fig. 5. **Nbs shift the equilibrium of Rho* from Meta II to Meta I/Lumi-like photoproducts.** **a,b** Comparison of ATR-FTIR difference spectra of bRho/Nbs (red curves) vs. reference spectra of pure Lumi (black curves, **a**) and Meta I spectrum (black curves, **b**). The reference spectra for Lumi (**a**) and Meta I bRho (**b**) were recorded at 200 K and at 240 K, respectively. Negative bands correspond with vibrations present in the ground state, and positive bands correspond with vibrations present in the respective intermediates. **c** Shift of Meta I/Meta II equilibrium for bRho* alone (minus sign) vs. bRho*/Nb2 complex (positive sign). Photoactivated bRho (t=0, black) was supplemented with Nb2 and the UV/Vis spectrum was recorded after a 4 h incubation on ice at pH 6.5 -8.5. **d** Shift of Meta I/Meta II equilibrium for bRho* in complex with Nb2, Nb7, Nb12, or Nb22 at pH 7.5. bRho* (t = 0 min, black) was incubated on ice with (positive sign) or without Nb (negative sign), followed by UV-Vis spectroscopy. **e** A478nm from the UV-Vis spectra in (**d**), plus two additional pH conditions were plotted together. Source data for panels **a-e** are provided as a Source Data file.

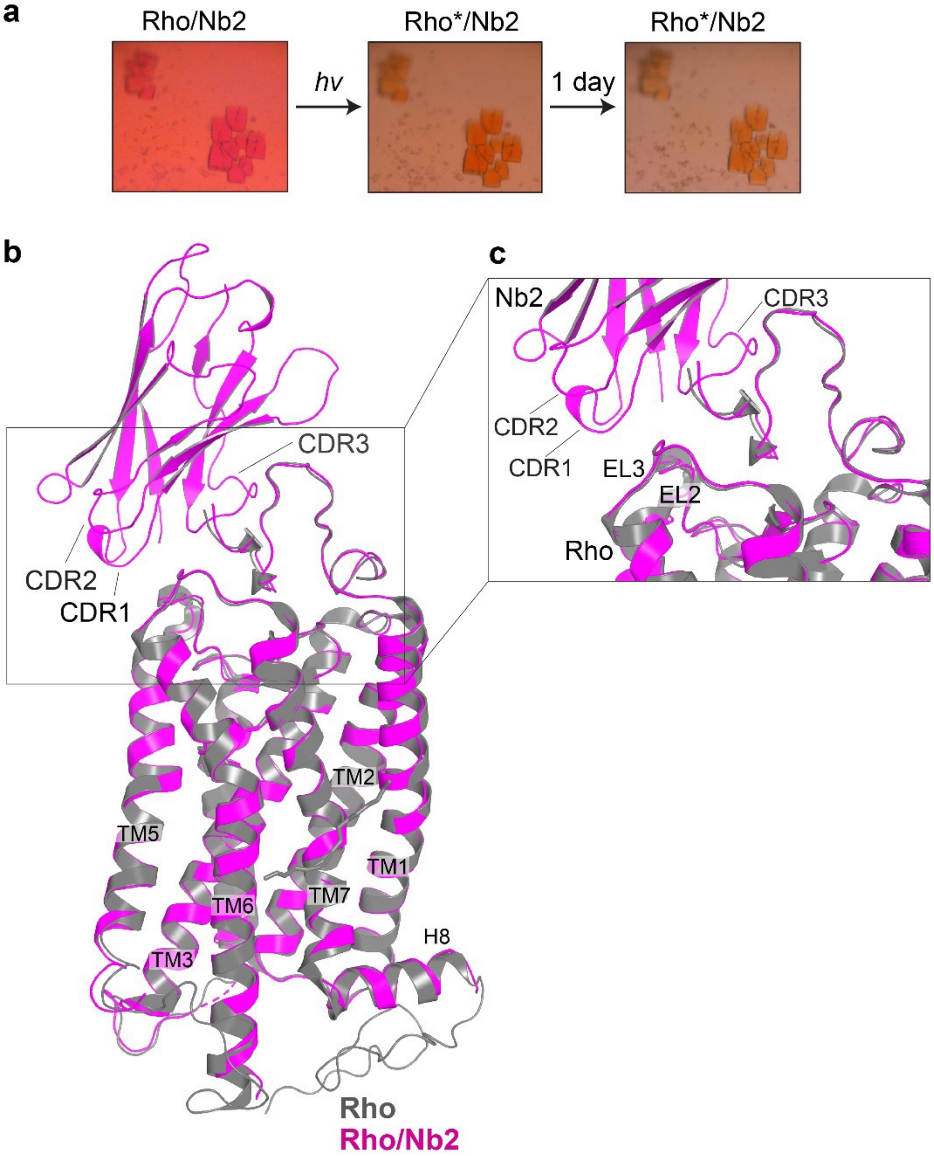

Supplementary Fig. 6. **Structure of bRho in complex with Nb2 *vs.* bRho alone. a** Crystals of ground- state bRho/Nb2 show color changes, turning red to orange after light exposure, indicating photoconversion of the Rho pigment. **b,c** Superposition of the crystal structures of ground-state bRho (grey, PDB ID 1U19 [https://doi.org/10.2210/pdb1U19/pdb]) and Rho/Nb2 complex (magenta) at 3.7 Å resolution. Glycans have been removed for clarity. Boxed region in (**b**) is enlarged in (**c**), showing the extracellular loops and the binding interface between bRho and Nb2.

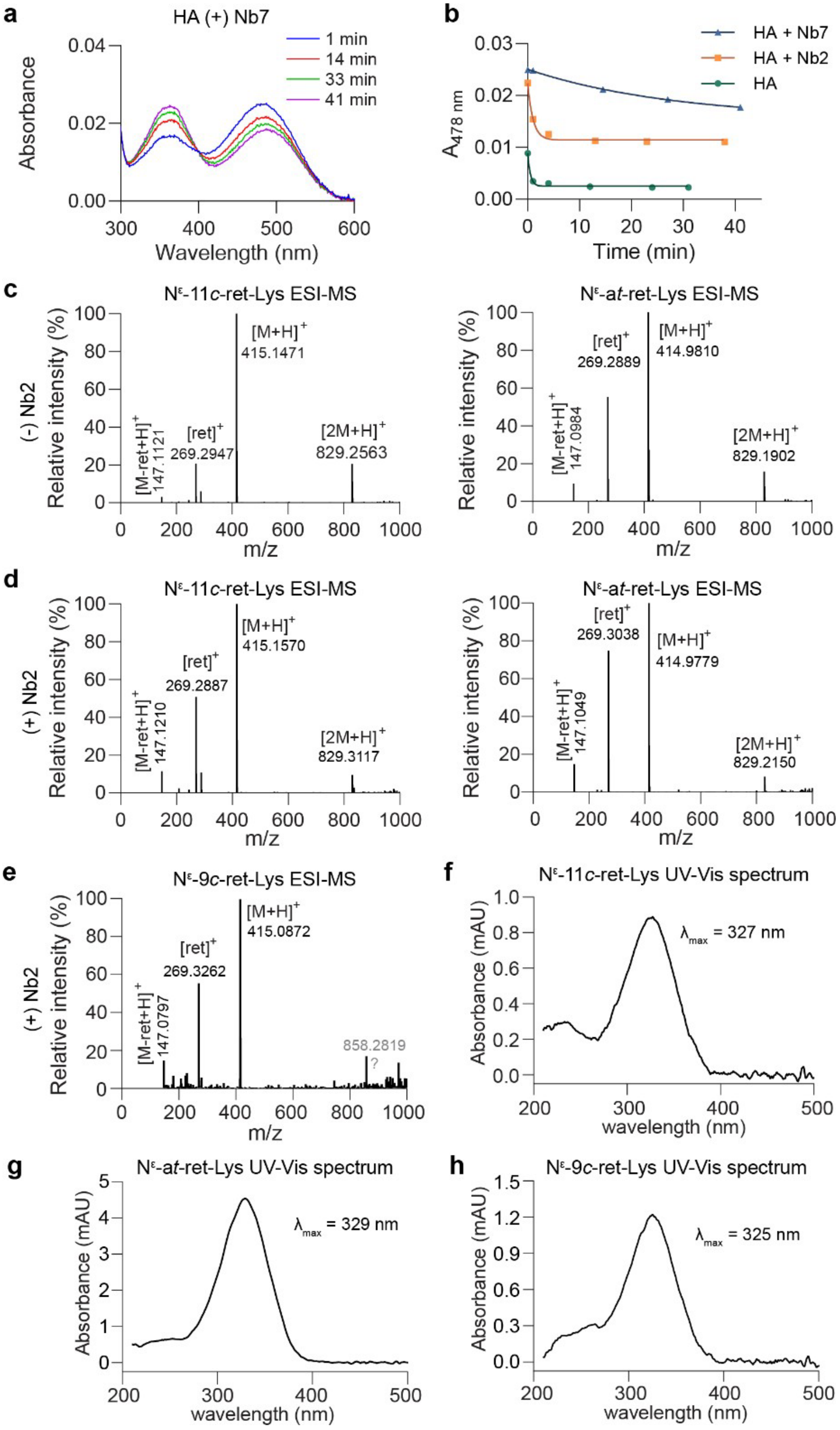
a
HA (+) Nb7
1 min
14 min
33 min
41 min
Absorbance
Wavelength (nm)
b
HA + Nb7
HA + Nb2
HA
A478 nm
Time (min)
c
Nε-11c-ret-Lys ESI-MS
(-) Nb2
Relative intensity (%)
[M+H]+
415.1471
[ret]+
269.2947
[M-ret+H]+
147.1121
[2M+H]+
829.2563
m/z
Nε-at-ret-Lys ESI-MS
[ret]+
269.2889
[M+H]+
414.9810
[M-ret+H]+
147.0984
[2M+H]+
829.1902
d
Nε-11c-ret-Lys ESI-MS
(+) Nb2
[M+H]+
415.1570
[ret]+
269.2887
[M-ret+H]+
147.1210
[2M+H]+
829.3117
Nε-at-ret-Lys ESI-MS
[ret]+
269.3038
[M+H]+
414.9779
[M-ret+H]+
147.1049
[2M+H]+
829.2150
e
Nε-9c-ret-Lys ESI-MS
(+) Nb2
[M+H]+
415.0872
[ret]+
269.3262
[M-ret+H]+
147.0797
858.2819
?
f
Nε-11c-ret-Lys UV-Vis spectrum
λmax = 327 nm
Absorbance (mAU)
wavelength (nm)
g
Nε-at-ret-Lys UV-Vis spectrum
λmax = 329 nm
h
Nε-9c-ret-Lys UV-Vis spectrum
λmax = 325 nm

Supplementary Fig. 7. **Schiff base hydrolysis is slowed by complex formation of bRho with Nbs. a** UV-Vis spectra of bRho*/Nb7 complex was measured after addition of 5 mM hydroxylamine (HA). **b** $A_{478\ nm}$ values from spectra in (**a**) and **Fig. 5c** were plotted using non-linear one phase decay. **c-d** Electrospray ionization (ESI) mass spectra from LC-MS of pronase proteolysis of $NaBH_4$-reduced bRho (**c**) or bRho/Nb2 complex (**d**); in the dark for the N^{ε}-11-*cis*-retinyl-Lys peak (left), and post-illumination for N^{ε}-all-*trans*-retinyl- Lys (right). **e** ESI-MS of Nε-9-cis-retinyl-Lys byproduct from photoisomerization of early photointermediates stabilized by Nb2. Characteristic source fragmentation of the retinyl-Lys precursor ion (415 m/z) was observed to yield the product retinyl cation (269 m/z) and Lys ion (147 m/z) from N^{ε}-11-*cis*-, N^{ε}-9-*cis*-, and N^{ε}-all-*trans*-retinyl-Lys. Representative UV-Vis absorption spectra for each N^{ε}-retinyl-Lys form collected during liquid chromatography: 11-*cis* (**f**), all-*trans* (**g**), and 9-*cis* (**h**). Source data for panels **a-h** are provided as a Source Data file.

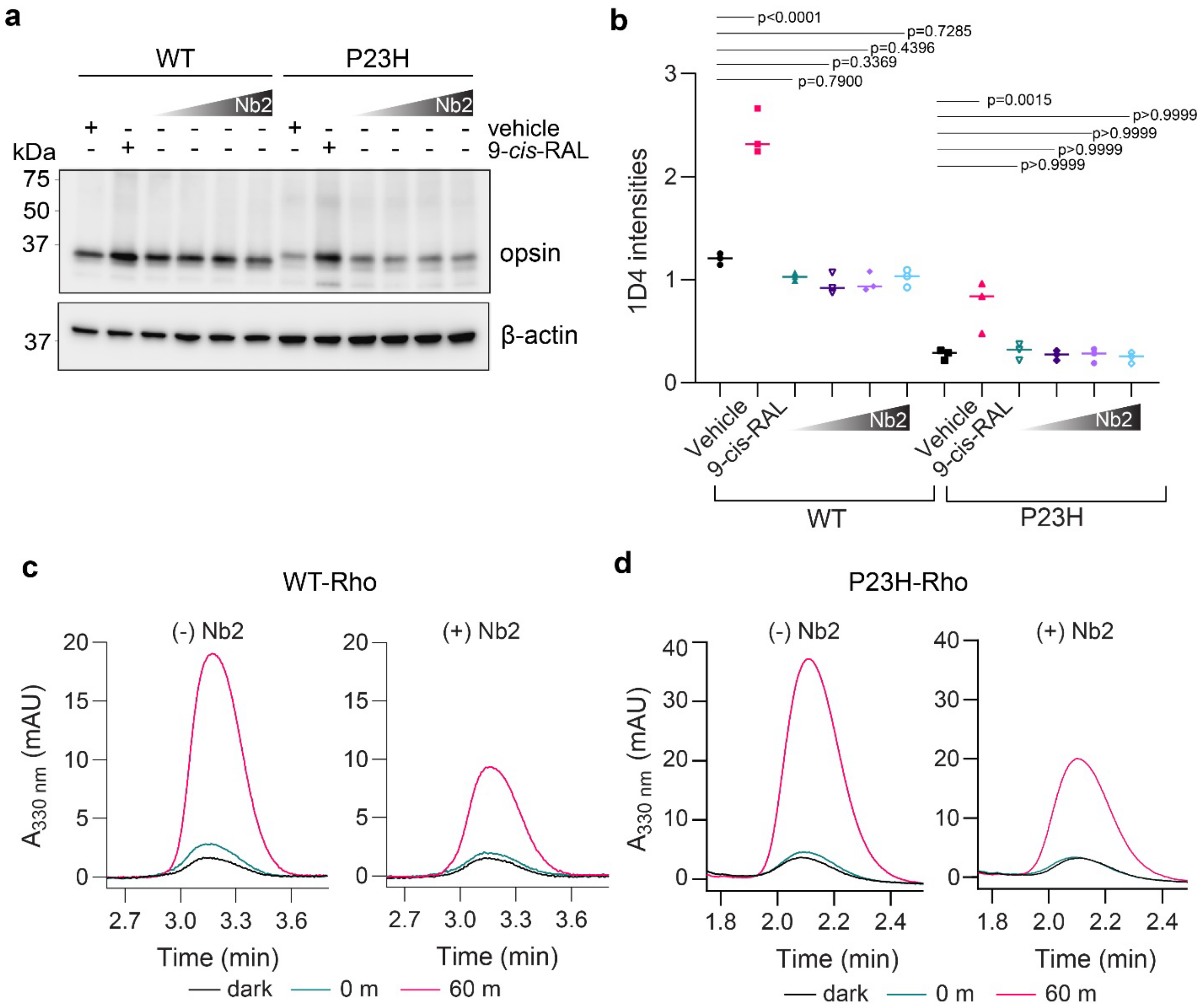

Supplementary Fig. 8. **Extracellular treatment with Nb2 does not restore the homeostasis of mutant- P23H-opsin. a** HEK293S cells overexpressing WT- or P23H-bOpsins were treated with 3-fold increasing concentrations of Nb2 ranging from 37 μM to 1 mM, followed by Western blotting. Treatment with 2.5 μM of 9-*cis*-retinal was used as a positive control. Lanes were loaded with either 7.5 μg of WT-bOpsin or 15 μg of P23H-bOpsin samples. **b** Relative 1D4 (opsin) levels were calculated after normalization to β-actin. One- way ANOVA was used to calculate the statistical significance of the difference between vehicle and treated cells (n = 3, biologically independent experiments). Horizontal bars represent means, and filled symbols represent individual data points. Adjusted *P*-values are indicated. **c,d** The alcohol solubilized fraction from $NaBH_4$-*i*PrOH treated WT- or P23H-bOpsin with or with Nb2 (from **Fig. 6d,f**) was analyzed by LC-MS for hydrolyzed free retinal at $A_{330\ nm}$, comparing ground-state (dark, black curve), immediately after illumination (0 min, green curve) and 1 h after illumination (60 min, red curve). Source data for panels **a-d** are provided as a Source Data file.

Supplementary Table

Supplementary Table 1. **X-ray crystallographic data collection and refinement statistics.**

	bRho-Nb2	bRho*- Nb2	apo-opsin-Nb2
**Data collection**			
Beamline	NECAT ID-E	NECAT ID-E	NECAT ID-E
Wavelength (Å)	0.97918	0.97918	0.97918
Space group	$P3_121$	$P3_121$	$P3_121$
Unit cell dimensions			
a, c (Å)	119.28, 227.12	120.29, 227.58	120.06, 230.93
Resolution (Å)	50-3.70 (3.92-3.70)*	50-4.25 (4.51-4.25)*	50-3.71 (3.94-3.71)*
Rmerge (%)	9.4 (283.1)	5.8 (355.0)	5.5 (365.9)
I / σI	10.4 (0.7)	14.3 (0.72)	15.8 (0.7)
$CC_{1/2}$ (%)	99.9 (37.8)	100 (26.4)	99.9 (22.1)
Completeness (%)	99.8 (100.0)	99.9 (100.0)	99.9 (100.0)
Redundancy	9.8 (10.1)	10.1 (10.7)	10.0 (9.9)
Wilson B-factor ($Å^2$)	195	280	217
**Refinement**			
Resolution (Å)	49.8-3.70	47.36-4.25	47.4-3.71
No. reflections	19,540 (1,029)‡	13,269 (697)‡	19,956 (1,033)‡
Rwork / Rfree (%)	28.1/33.2	27.9/29.5	29.2/29.5
No. atoms	6,872	6,760	6,832
Protein	6,629	6,557	6,629
Glycans	203	203	203
Retinylidene	40	-	-
B-factors ($Å^2$)	218	304	239
Protein	218	304	239
Glycans	230	318	246
Retinylidene	201	-	-
R.M.S. deviations			
Bond lengths (Å)	0.002	0.004	0.002
Bond angles (°)	0.718	1.045	0.670
Ramachandran plot			
Favored (%)	93.3	91.6	92.7
Number disallowed	0	0	0
PDB accession codes	8FCZ	8FD1	8FD0

* Highest-resolution shell is shown in parentheses.

‡ Number of reflections used for cross-validation

Supplementary References

1. Sievers F., *et al.* Fast, scalable generation of high-quality protein multiple sequence alignments using Clustal Omega. *Mol. Syst. Biol.* **7**, 539 (2011).

2. Letunic I., Bork P. Interactive Tree Of Life (iTOL) v5: an online tool for phylogenetic tree display and annotation. *Nucleic Acids Res.* **49**, W293-W296 (2021).

3. Sievers F., Higgins D. G. The Clustal Omega Multiple Alignment Package. *Methods Mol. Biol.* **2231**, 3-16 (2021).

4. Gouet P., Courcelle E., Stuart D. I., Metoz F. ESPript: analysis of multiple sequence alignments in PostScript. *Bioinformatics* **15**, 305-308 (1999).
