# Supplementary material for: Structural basis for the allosteric modulation of rhodopsin by nanobody binding to its extracellular domain
Source: Nat Commun. 2023 Aug 25;14:5209. doi: 10.1038/s41467-023-40911-9 (PMC10457330; doi:10.1038/s41467-023-40911-9)
Supplement: Supplementary file 3 — Reporting Summary [file 41467_2023_40911_MOESM3_ESM.pdf]

nature portfolio

Corresponding author(s): K. Palczewski, P.D. Kiser, K. Katayama

Last updated by author(s): May 8, 2023

Reporting Summary

Nature Portfolio wishes to improve the reproducibility of the work that we publish. This form provides structure for consistency and transparency in reporting. For further information on Nature Portfolio policies, see our Editorial Policies and the Editorial Policy Checklist.

Statistics

For all statistical analyses, confirm that the following items are present in the figure legend, table legend, main text, or Methods section.

n/a	Confirmed	
☐	☒	The exact sample size (*n*) for each experimental group/condition, given as a discrete number and unit of measurement
☐	☒	A statement on whether measurements were taken from distinct samples or whether the same sample was measured repeatedly
☐	☒	The statistical test(s) used AND whether they are one- or two-sided<br>*Only common tests should be described solely by name; describe more complex techniques in the Methods section.*
☒	☐	A description of all covariates tested
☒	☐	A description of any assumptions or corrections, such as tests of normality and adjustment for multiple comparisons
☐	☒	A full description of the statistical parameters including central tendency (e.g. means) or other basic estimates (e.g. regression coefficient) AND variation (e.g. standard deviation) or associated estimates of uncertainty (e.g. confidence intervals)
☐	☒	For null hypothesis testing, the test statistic (e.g. *F*, *t*, *r*) with confidence intervals, effect sizes, degrees of freedom and *P* value noted<br>*Give P values as exact values whenever suitable.*
☒	☐	For Bayesian analysis, information on the choice of priors and Markov chain Monte Carlo settings
☒	☐	For hierarchical and complex designs, identification of the appropriate level for tests and full reporting of outcomes
☒	☐	Estimates of effect sizes (e.g. Cohen's *d*, Pearson's *r*), indicating how they were calculated

Our web collection on statistics for biologists contains articles on many of the points above.

Software and code

Policy information about availability of computer code

Data collection

no new software was developed for data collection.

Data analysis

no new software was developed for data analysis. Following software were used for data analysis.
XDS: v20210323 and v20220220
BALBES: v1.0.0
Phaser: v2.8.3
Refmac5: v5.8.0352
Coot: v0.9.8
Privateer: vMKIV : 06/02/2021
Molprobity server: v4.5
wwPDB validation pipeline: v2.31.3

For manuscripts utilizing custom algorithms or software that are central to the research but not yet described in published literature, software must be made available to editors and reviewers. We strongly encourage code deposition in a community repository (e.g. GitHub). See the Nature Portfolio guidelines for submitting code & software for further information.

Data

Policy information about availability of data

All manuscripts must include a data availability statement. This statement should provide the following information, where applicable:

- Accession codes, unique identifiers, or web links for publicly available datasets
- A description of any restrictions on data availability
- For clinical datasets or third party data, please ensure that the statement adheres to our policy

The data that support the findings of this study are available within the Source Data file included with this manuscript. Biological materials used in the findings of this manuscript are available from the corresponding author upon reasonable request. The crystal structures and their associated diffraction data generated in this study are available in the Protein Data Bank (PDB) under accession codes:
8FCZ [https://www.rcsb.org/structure/unreleased/8FCZ] (bRho/Nb2)
8FD1 [https://www.rcsb.org/structure/unreleased/8FD1] (bRho*/Nb2)
8FDO [https://www.rcsb.org/structure/unreleased/8FDO] (opsin/Nb2)

Other crystals structures used in this study are available from the PDB under accession codes: 1L9H (bRho), 4BEL (Bace2), 1U19 (bRho), and 5TE3 (Bos taurus opsin)

Human research participants

Policy information about studies involving human research participants and Sex and Gender in Research.

Reporting on sex and gender	n/a
Population characteristics	n/a
Recruitment	n/a
Ethics oversight	n/a

Note that full information on the approval of the study protocol must also be provided in the manuscript.

Field-specific reporting

Please select the one below that is the best fit for your research. If you are not sure, read the appropriate sections before making your selection.

☒ Life sciences ☐ Behavioural & social sciences ☐ Ecological, evolutionary & environmental sciences

For a reference copy of the document with all sections, see nature.com/documents/nr-reporting-summary-flat.pdf

Life sciences study design

All studies must disclose on these points even when the disclosure is negative.

Sample size	No sample size calculations were performed, as we performed three biologically independent replicates to confirm reproducibility. Due to the low variability observed between samples, we considered this to be sufficient. We used sample size of three for 3 individual repeats for mutagenesis analysis, tryptophan quenching assay and rescue assay of misfolded rhodopsin. FTIR spectra were performed on 3 different samples each time. For the rest of the data, we carried out 3 independent experiments with similar results and show one representative experiment unless it is noted otherwise in the figure legends.
Data exclusions	No data were excluded.
Replication	Three biologically independent experiments were successfully replicated. When mammalian cells were used, different frozen cell stocks were prepared for each biologically independent experiment, performed in three different times.
Randomization	The areas used for imaging were selected randomly. Randomization is overall not relevant in this study since results are often analyzed by biochemical assays such as Western blot in which samples were loaded in a specific order for a comparison.
Blinding	Blinding was not relevant to this study. Investigators were not blinded to group allocation because group allocation was not involved in our study.

Reporting for specific materials, systems and methods

We require information from authors about some types of materials, experimental systems and methods used in many studies. Here, indicate whether each material, system or method listed is relevant to your study. If you are not sure if a list item applies to your research, read the appropriate section before selecting a response.

Materials & experimental systems

n/a	Involved in the study
☐	☒ Antibodies
☐	☒ Eukaryotic cell lines
☒	☐ Palaeontology and archaeology
☐	☒ Animals and other organisms
☒	☐ Clinical data
☒	☐ Dual use research of concern

Methods

n/a	Involved in the study
☒	☐ ChIP-seq
☒	☐ Flow cytometry
☒	☐ MRI-based neuroimaging

Antibodies

Antibodies used	Anti-β-actin 13E5 antibody (Cell Signaling, catalog no. 4970), anti-Rho 1D4 antibody (obtained in-house from hybridoma cells), Alexa Fluor 647-conjugated goat anti-mouse IgG antibody (Invitrogen, catalog no. A21236). Twenty seven nanobodies against bovine rod outer segments were raised by llama immunization.
Validation	Anti-β-actin 13E5 antibody was validated by the manufacturer for the species reactivity with human, mouse, rabbit, monkey, bovine and pig beta-actin proteins and for the applications such as Western blot, immunoprecipitation, immunohistochemistry, Chip, immunofluorescece and Flow cytometry.<br>For 1D4 antibody, validation is described in a journal, published in 1984 by MacKenzie et al. under the title of "Localization of binding sites for carboxyl terminal specific anti-rhodopsin monoclonal antibodies using synthetic peptides. " (Biochemistry 1984, 23(26):6544-9). The 1D4 antibody reacts with mouse, rat, human and cow Rhodopsin and is suitable for immunohistochemistry, immunofluorescent, co-immunoprecipitation and Western blot.<br>For anti-rhodopsin nanobodies, the formation of stable nanobody/bovine rhodopsin complexes was confirmed by co-immunoprecipitation and blue native PAGE in this study.

Eukaryotic cell lines

Policy information about cell lines and Sex and Gender in Research

Cell line source(s)	HEK293S (catalog no. CRL-3022), NIH3T3 (catalog no. CRL-1658)and AMPHO (catalog no. CRL-3213) cells were purchased from ATCC (VA, USA). All three cell lines require Biosafety level 2 (BSL-2).
Authentication	Stable cell lines established in this manuscript were not authenticated.
Mycoplasma contamination	All cell lines were tested negative for micoplasma contamination.
Commonly misidentified lines (See ICLAC register)	No commonly misidentified cell lines were used in the study.

Animals and other research organisms

Policy information about studies involving animals; ARRIVE guidelines recommended for reporting animal research, and Sex and Gender in Research

Laboratory animals	6-8-week-old C57BL6/J mice were purchased from the Jackson Laboratory (Jackson Laboratory; Bar Harbor; strain # 000664). Mice were housed with a 14/10-hour light/dark cycle, with free access to food and water. Breeding and experimental rooms were maintained at an ambient temperature of 20-26°C, and a humidity of 40–60%. One female, 6-years-and-7-moths-old Llama glama was use for immunization with bovine rod outer segments.
Wild animals	No wild animals were used in this study.
Reporting on sex	Mice of both sexes were used. A single female llama was used. No sex-related effects are expected in this study.
Field-collected samples	No field-collected samples were used in this study.
Ethics oversight	Mouse experiments were approved by the Institutional Animal Care and Use Committees (IACUC) of the University of California, Irvine. Llama immunization was performed in accordance with institutional guidelines, following experimental protocol reviewed and approved by the Vrije Universiteit Brussel Ethical Committee for Animal experiments

Note that full information on the approval of the study protocol must also be provided in the manuscript.
