# Supplementary material for: Structural basis for the allosteric modulation of rhodopsin by nanobody binding to its extracellular domain
Source: Nat Commun. 2023 Aug 25;14:5209. doi: 10.1038/s41467-023-40911-9 (PMC10457330; doi:10.1038/s41467-023-40911-9)

Fig. 1g, upper

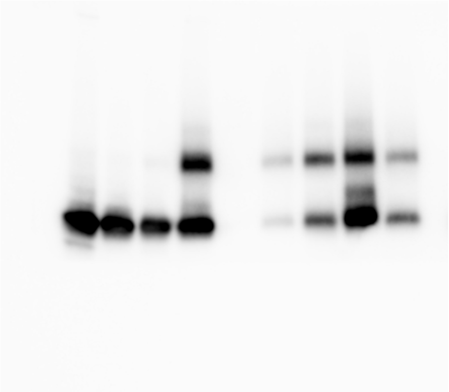

Fig. 1i, upper

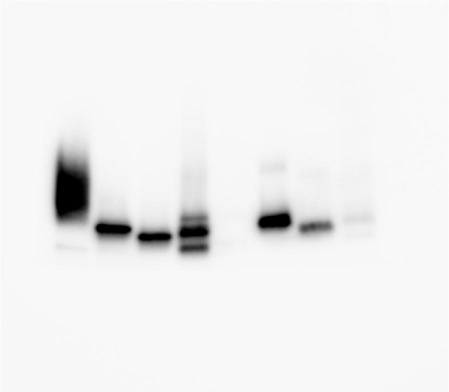

Fig. 1g, lower

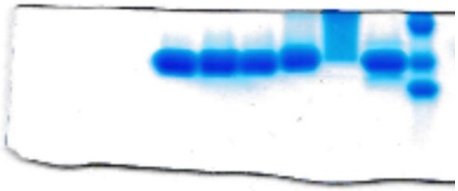

Fig. 1i, lower

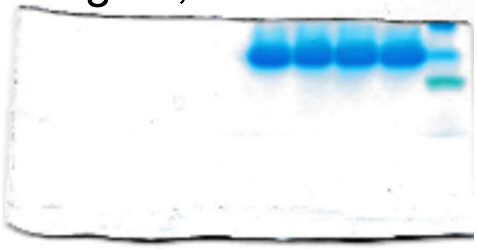

Fig. 6a, upper

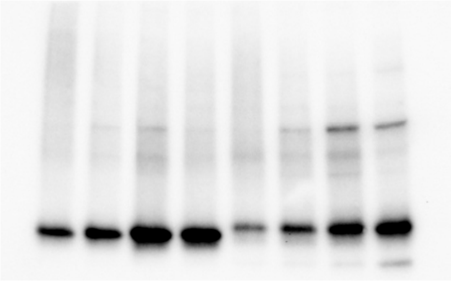

Fig. 6a, lower

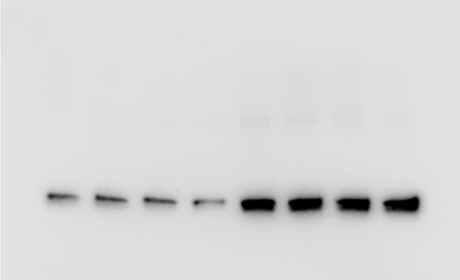

Fig. 6c

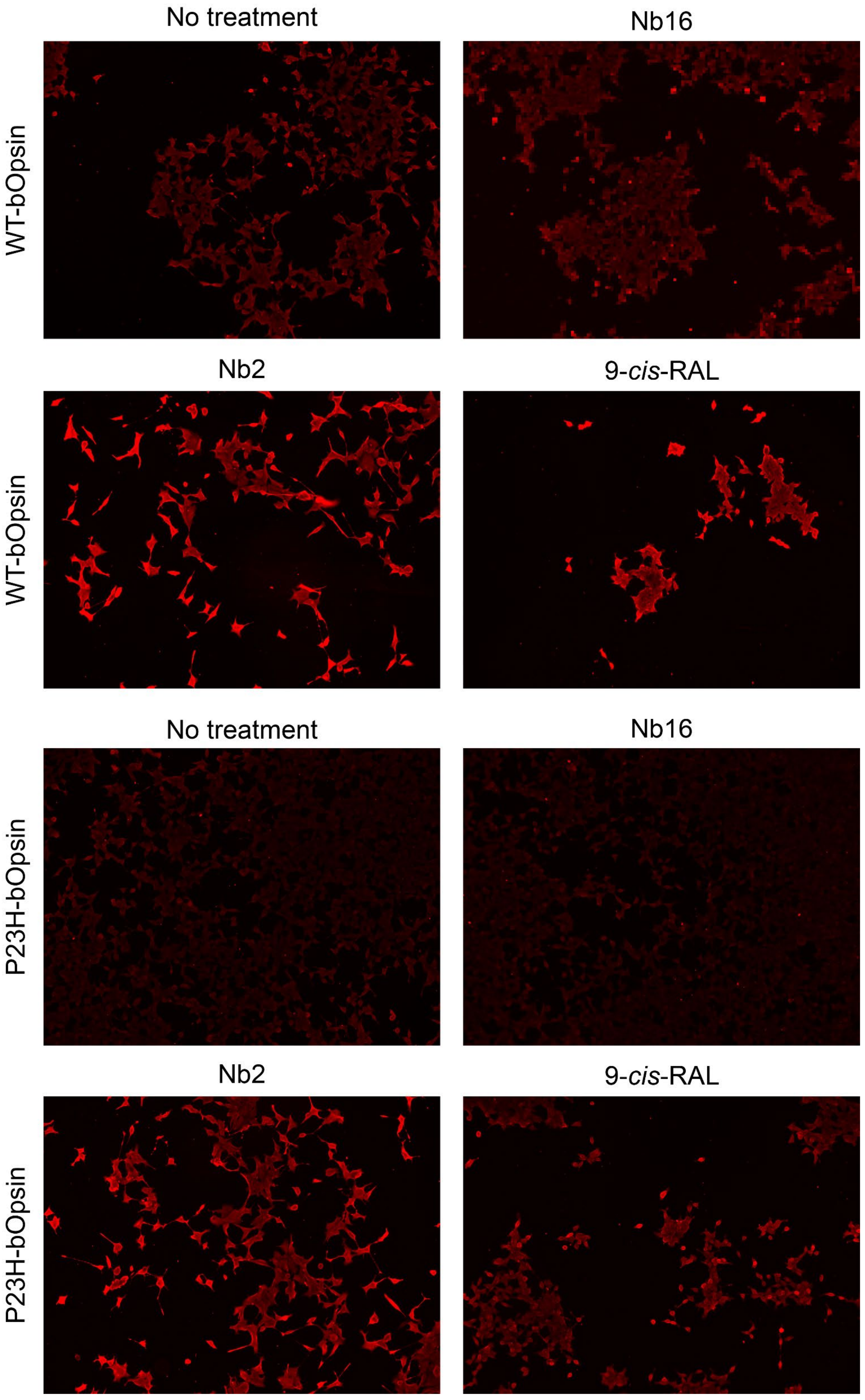
No treatment
Nb16
WT-bOpsin
Nb2
9-*cis*-RAL
WT-bOpsin
No treatment
Nb16
P23H-bOpsin
Nb2
9-*cis*-RAL
P23H-bOpsin

Supplementary Fig. 1a, light

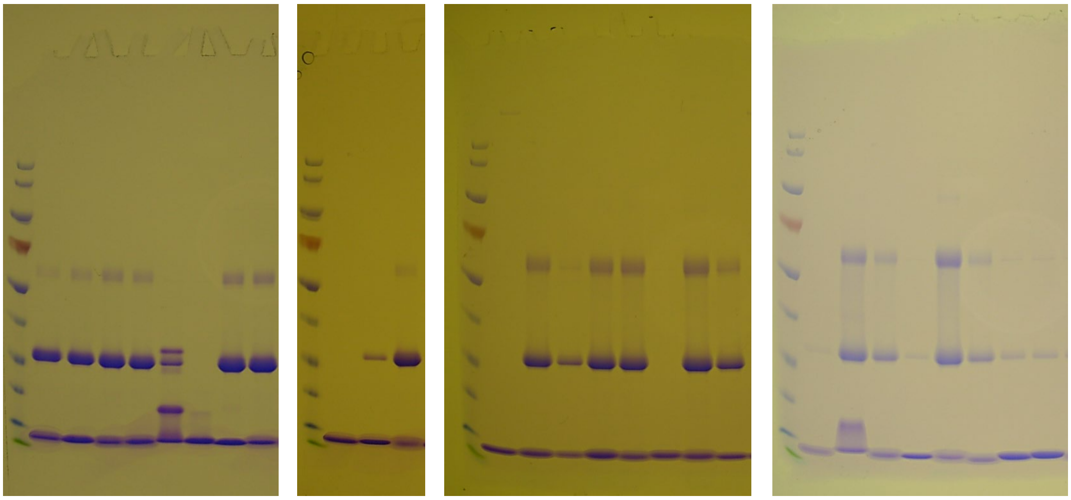

Supplementary Fig. 1a, dark

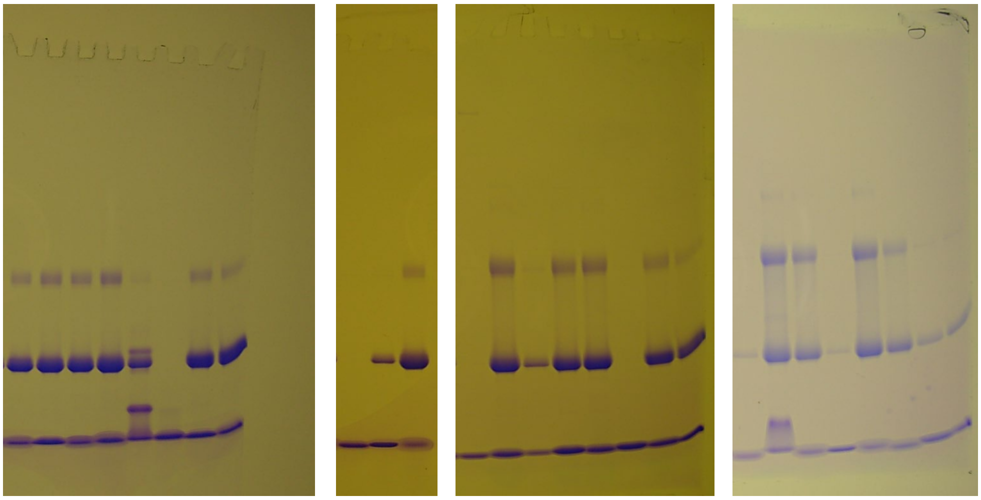

Supplementary Fig. 1b, Nb2

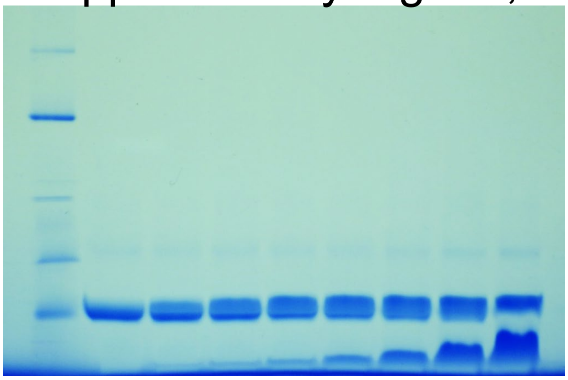

Supplementary Fig. 1b, Nb7

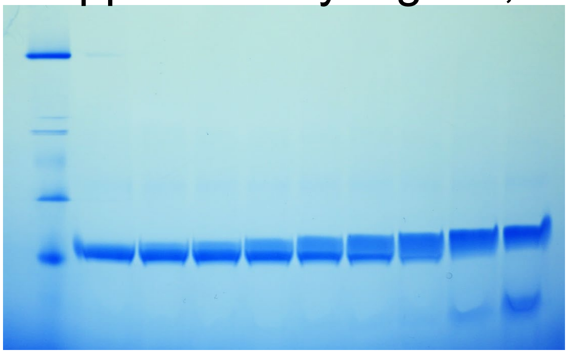

Supplementary Fig. 1b, Nb12

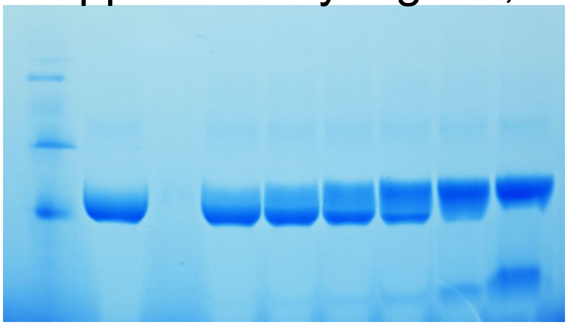

Supplementary Fig. 1b, Nb22

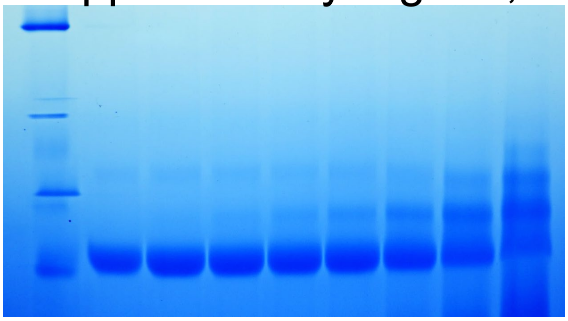

Supplementary Fig. 1c, Dark

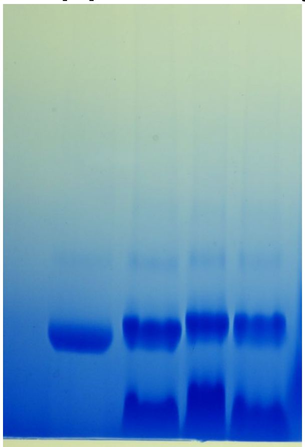

Supplementary Fig. 1c, Light

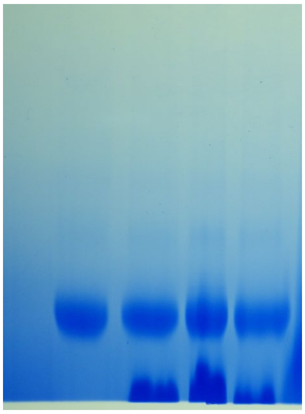

Supplementary Fig. 3a, Left, western

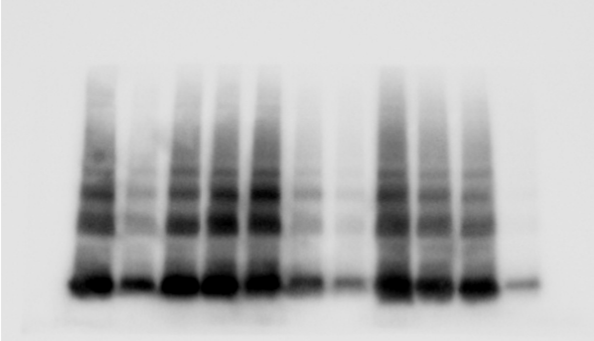

Supplementary Fig. 3a, Left, Coomassie

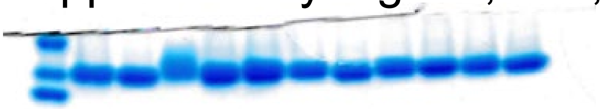

Supplementary Fig. 3a, right, Western blot

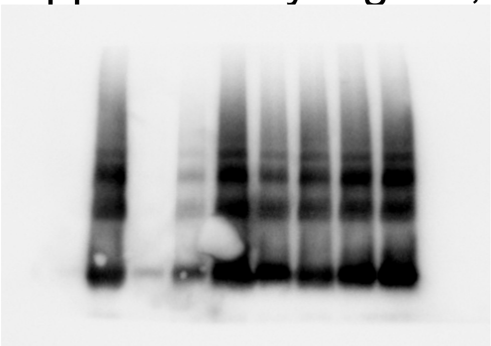

Supplementary Fig. 3a, right, Coomassie

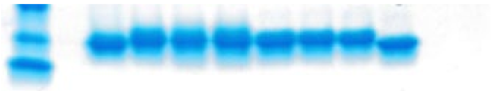

Supplementary Fig. 3c

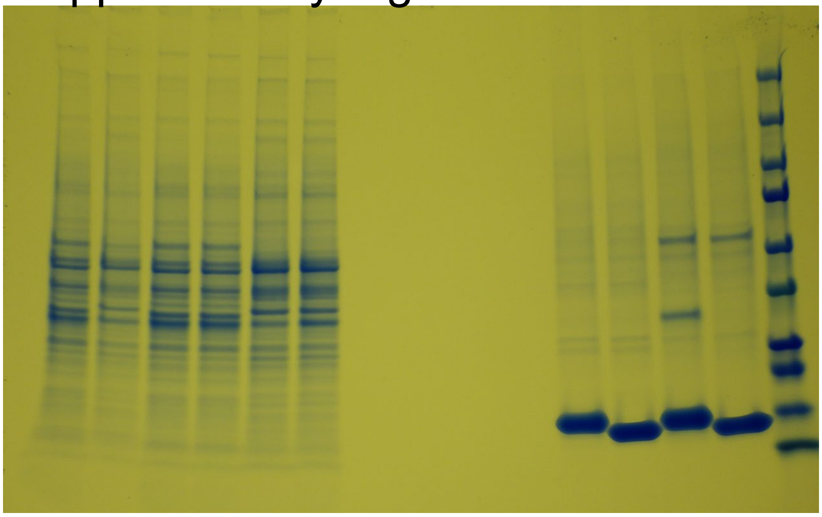

Supple Figure 8a, upper

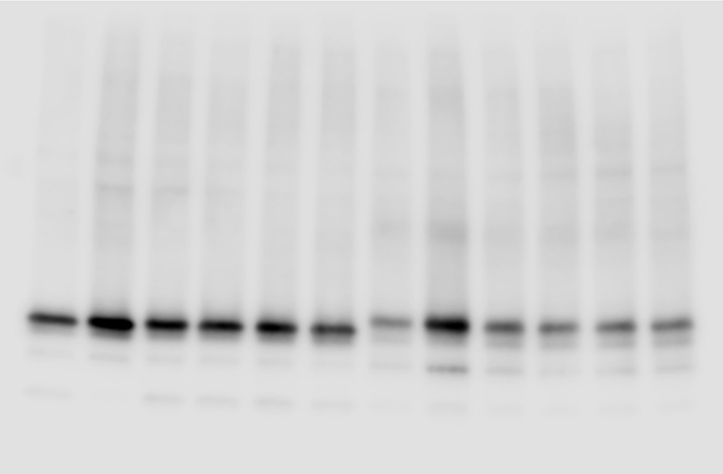

Supple Figure 8a, lower

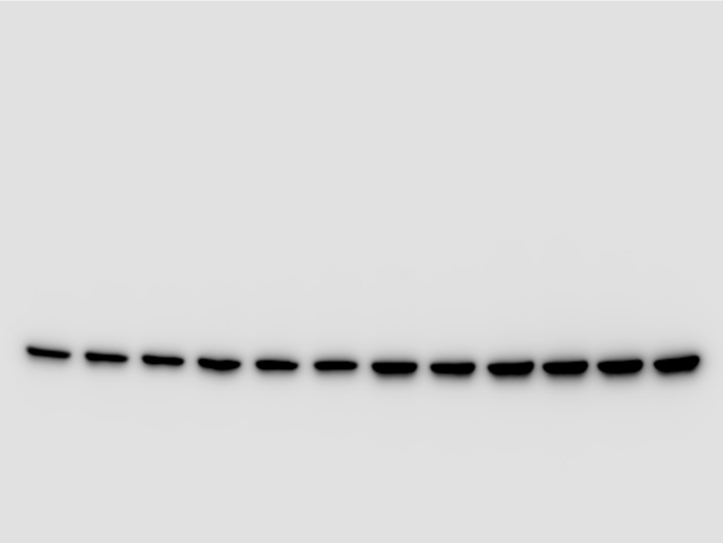

Supplement: Supplementary file 4 — Source Data [file 41467_2023_40911_MOESM4_ESM.zip › Source Data-gels-blots.pdf]
